# The PLOS ONE Synthetic Biology Collection: Six Years and Counting

**DOI:** 10.1371/journal.pone.0043231

**Published:** 2012-08-15

**Authors:** Jean Peccoud, Mark Isalan

**Affiliations:** 1 Virginia Bioinformatics Institute, Virginia Tech, Blacksburg, Virginia, United States of America; 2 Center for Systems Biology of Engineered Tissues, Institute for Critical Technologies and Applied Science, Virginia Tech, Blacksburg, Virginia, United States of America; 3 EMBL/CRG Systems Biology Research Unit, Centre for Genomic Regulation (CRG) and UPF, Barcelona, Spain; Nanyang Technological University, Singapore

## Abstract

Since it was launched in 2006, PLOS ONE has published over fifty articles illustrating the many facets of the emerging field of synthetic biology. This article reviews these publications by organizing them into broad categories focused on DNA synthesis and assembly techniques, the development of libraries of biological parts, the use of synthetic biology in protein engineering applications, and the engineering of gene regulatory networks and metabolic pathways. Finally, we review articles that describe enabling technologies such as software and modeling, along with new instrumentation. In order to increase the visibility of this body of work, the papers have been assembled into the PLOS ONE Synthetic Biology Collection (www.ploscollections.org/synbio). Many of the innovative features of the PLOS ONE web site will help make this collection a resource that will support a lively dialogue between readers and authors of PLOS ONE synthetic biology papers. The content of the collection will be updated periodically by including relevant articles as they are published by the journal. Thus, we hope that this collection will continue to meet the publishing needs of the synthetic biology community.

## Introduction

Synthetic biology is an emerging transdisciplinary field at the intersection between many engineering and scientific disciplines such as biology, chemical engineering, chemistry, electrical engineering, or computer science. The scientific milestone that inspired the development of synthetic biology is often regarded as the description of two artificial gene networks in the same issue of Nature in 2000 [1], [2]. However, the year 2004 marks the emergence of synthetic biology as a scientific community. This is the year of the first synthetic biology conference, the first iGEM competition –where students compete to build biological systems (http://igem.org/) ― and the creation of the synthetic biology page on Wikipedia. Two years later, the first issue of PLOS ONE included two synthetic biology articles [3], [4], marking the beginning of a trend. Since then, PLOS ONE has published a large number of articles covering all aspects of the field. Synthetic biologists resolutely push the limits of their specialties in ways that few established journals have been able to appreciate. Since the result is often more “how to build something that works” rather than primary biological insight, the papers can be hard to place in classical journals. Many synthetic biology authors have benefited from the innovative PLOS ONE editorial policy to publish scientifically sound research, irrespective of its anticipated significance.

The purpose of this article is to introduce the PLOS ONE Synthetic Biology Collection (www.ploscollections.org/synbio/). The collection highlights selected synthetic biology articles published in PLOS ONE since 2006, putting them together in one place for easy perusal. The website is intended to be a growing resource that will be updated regularly.

We review the collection here by organizing it into some broad categories: DNA synthesis and assembly, Biological parts, Protein engineering, Networks and pathways, Synthetic life, Software and modeling, and Instruments. The classification is our own; since many synthetic biology papers cited in this review span more than one category, it was sometimes difficult to assign them to one category rather than another. Nonetheless, this structure should aid in navigating the 50+ papers currently included in the collection.

## Summary of Papers Included in the Collection

### DNA Synthesis and Assembly

Synthetic biology projects often begin with the assembly of complicated, multi-component gene constructs. Therefore, both DNA assembly and cloning technologies are critical enablers of synthetic biology. Not surprisingly, many recent PLOS ONE papers propose methods to improve the efficiency of the fabrication step of synthetic biology projects. For example, Golden Gate Cloning [5] is a one-step DNA assembly protocol that can join at least nine distinct DNA fragments into one plasmid vector. The technique employs type IIs restriction enzymes that cut DNA at some distance from their cognate DNA-binding site, thus allowing flexibility and uniqueness in the compatible sticky ends that are generated. A related technique is GoldenBraid Assembly [6], that also uses type IIs restriction enzymes, but applies them iteratively to standardized DNA parts (see the ‘Biological parts’ section below). This allows the indefinite growth of reusable gene modules. Similarly, type IIs restriction enzymes have been used to make a hierarchical modular cloning system aimed at making eukaryotic multigene constructs [7].

‘One-pot’ assembly and cloning systems are being developed by many groups, and the ideal systems use as few standardized components as possible. Circular polymerase extension cloning (CPEC) fits into this category, using a single polymerase to assemble and clone multiple inserts with any vector, in a one-step *in vitro* reaction [8]. Alternatively, successive hybridization assembling (SHA) also employs a single reaction *in vitro* [9].

As well as cloning one desired multi-component construct, many projects require degenerate cloning or mutagenesis to make combinatorial libraries of gene variants. The OmniChange technique, which simultaneously saturates five independent codons, has therefore been developed to generate full-length gene libraries with 5 degenerate NNK-codons while avoiding PCR-amplification [10]. Large libraries of genetic sequences can be derived from oligonucleotides synthetized in a microarray, and later pooled in libraries from which more complex sequences can be derived [11]. By combining linear DNA amplification and PCR, DNA libraries with hundreds to thousands of members can be synthesized.

PCR methods themselves can have certain limitations, such as difficulties in amplifying GC-rich DNA targets. One study optimized polymerase chain assembly (PCA) and ligase chain reaction (LCR) methods for the construction of two GC-rich gene fragments implicated in tumorigenesis, IGF2R and BRAF [12]. They found that LCR was superior and benefited from the addition of DMSO and betaine.

The many synthesis and assembly methods presented in the collection can be combined to streamline the fabrication steps of synthetic biology projects, by producing collections of standardized biological parts. Standard parts are themselves a distinctive feature of synthetic biology, as reviewed below.

### Biological Parts

The Registry of Standard Biological Parts (www.partsregistry.org), based on the original vision of Tom Knight, is providing a rich collection of components for synthetic biology projects. Several articles in the PLOS ONE collection reflect the importance of this resource. For example, a global analysis of the Registry clone collection [13] helped identify certain discrepancies between the sequences recorded in the database and the physical sequences of some clones in the collection. These results prompted a change in the quality control of the submissions to the Registry that has greatly improved the overall quality of the collection. Moreover, the analysis of parts usage patterns led to organizational guidelines that may help design and manage these new types of scientific resources. As most parts in the registry are for prokaryotes, a eukaryotic collection of 52 parts was developed and is available for distribution [14]. This includes multiple cloning sites (MCS), common protein tags, protein reporters and selection markers, amongst others. Furthermore, most of the parts were designed in a format to allow fusions that maintain the reading frame.

As well as standardized coding regions, synthetic biology projects require well-characterized promoters to achieve desired expression strengths. In one study, a single yeast promoter was mutated to make a fine-graded output range promoter library [15]. Transcription Activator-Like Orthogonal Repressors were then developed synthetically to control expression of these promoters in an orthogonal manner. Such orthogonality or ‘non-cross-reactivity’ is necessary for engineering larger synthetic gene circuits that do not interfere with the physiology of the biological chassis in which they operate. Mammalian synthetic promoters have also been developed by analyzing motifs found in highly active human promoters. Thus, by modulating the amount of sequences rich in GC and CpGs, custom designed promoters were obtained [16].

Finally, entirely *de novo* parts that are found nowhere in nature have been engineered to slot into biological systems. Using *E. coli* lacking conditionally essential genes, entirely new functional proteins were obtained from scaffolds of randomized 4-helix bundles, rescuing stalled growth [17]. Similarly, a synthetic ATP-binding protein, evolved entirely from non-natural sequences, was expressed in *E. coli*, altering the levels of intracellular ATP [18]. Protein engineering approaches are thus a potential source of many new parts, as well as forming a branch of synthetic biology in their own right.

### Protein Engineering

Protein engineering can take many forms, from directed evolution methods to protein design. The PLOS ONE Synthetic Biology Collection includes a wide range of studies in this broad field.

Phage display is one of the classic tools of protein engineering, allowing combinatorial libraries of randomized proteins to be selected from the surface of bacteriophages. Phage display was used to generate a new class of binding proteins targeted to the pointed-end of actin [19]. These proteins, called synthetic antigen binders (sABs), were based on an antibody-like scaffold where sequence diversity is introduced into the binding loops using a new “reduced genetic code” phage display library.

An example of targeted protein design was the design of a dual reporter, Gemini [20]. Here, β-galactosidase (β-gal) α-fragment was fused to GFP, resulting in increased β-gal activity and some decrease in GFP sensitivity. GFP was also modified in a study where the ten proline residues of enhanced green fluorescent protein (EGFP) were replaced by (4*R*)- and (4*S*)-fluoroprolines (FPro) [21]. In this way, protein folding and stability could be tuned.

A promising advance in the field of engineering custom sequence-specific DNA-binding proteins is the use of Transcription Activator-Like (TAL) proteins. Modular TAL units specify A, C, G or T and can be concatenated to make long designer DNA-binding domains. Thus, Golden TAL Technology [22] has adapted Golden Gate Cloning [5] for engineering new TAL proteins. These were shown to function in human and plant cells and to target activation of both exogenous and endogenous genes, after fusion with a VP16 activation domain.

As well as single proteins, entire pathways can nowadays be engineered. Computational redesign was used to create new periplasmic binding proteins in plants, to act as biosensors in combination with a histidine kinase signaling cascade [23]. This resulted in transcription factor activation and ‘de-greening’ of plants in response to small-molecule stimuli. As can be seen from this example and the ones below, the move from single protein engineering to network engineering is one of the main driving forces in synthetic biology.

### Networks and Pathways

One of the first, and now most-cited, synthetic biology papers in PLOS ONE was the study on fitness-induced attractor selection [3]. Here, a synthetic mutual inhibition gene network was built in *E. coli*, with two states, green (GFP) and red (RFP), that were mutually exclusive. By attaching a fitness pressure to one of the states (i.e. a gene required for growth in the absence of glutamine), the authors demonstrated that the cells switched stochastically into the fittest state, restoring growth. In other words, by changing to a glutamine-free medium, the red cells switched to green, even in the absence of formal signaling machinery. This work has important messages for potential new mechanisms in gene regulation, where underlying fitness pressures can ultimately determine how much a gene is expressed, simply according to need.

Other small bacterial networks have been built to include a heritable sequential memory switch, using the *fim* and *hin* inversion recombination systems [24], and an *E. coli* strain for use as a ‘chemical recording device’ [25]. In the latter, the authors created a synthetic chemically sensitive genetic toggle switch to activate appropriate fluorescent protein indicators (GFP, RFP) and along with a cell division inhibitor (*minC*). Moving to yeast, one example of network engineering was the reconstruction of a human p53-Mdm2 negative feedback module in *S. cerevisiae* [26]. In this example, many aspects of p53 regulation in mammals were maintained, such as Mdm2-dependent targeting of p53 for degradation, sumoylation at lysine 386 and further regulation of this process by p14ARF. In mammalian systems, a synthetic tetracycline regulator positive feedback loop was stably integrated and yielded a bimodal expression response because such cells can only be “OFF” or “ON” [27].

One unusual work in synthetic biology aimed to rewire and control cell shape in yeast, by changing the inputs into the α-factor pathway [28]. This pathway can give rise to multiple mating projections, upon prolonged activation. The authors tested genetic manipulations that ultimately gave rise to single or multiple projections, in the absence of the natural input, α-factor.

A group of papers in the collection explore ‘synthetic ecology’, where consortia of different cells interact to give patterns at a population level. For example, by engineering two strains of *E. coli*, one study was able to achieve synthetic biofilms with spatial self-organization [29]. The consortia achieved defined layered structures and had unexpected growth advantages. A second paper describes a systems composed of two quorum-sensing signal transduction circuits that allowed the authors to build a synthetic ecosystem where the population dynamics could be tuned by varying the environmental signals [30]. Third, quorum components were also used in a study which generated robust but unexpected oscillations in *E. coli* by building synthetic suicide circuits [31]. In fact, the quorum components proved to be unnecessary to achieve oscillations: there was a density-dependent plasmid amplification that gave rise to population-level negative feedback, ultimately resulting in the cycles. As in other areas of synthetic biology, the process of building systems often leads to surprises which can result in useful new engineering tools, or to a better understanding of the underlying biological processes [32].

Pathway engineering for the production of useful chemical or product synthesis is a major field within synthetic biology. For example, an engineered yeast that efficiently secretes penicillin was built by transplanting synthesis pathway components into a host that is more suited for pharmaceutical production [33]. Artemisinin derivatives are key components of malaria therapies and their synthesis is a high-profile goal of synthetic biology because extraction from slow-growing plants currently limits supply. Consequently, one study achieved high-level production of an artemisinin precursor in *E. coli* [34]. Another striking synthesis paper demonstrates a synthetic enzymatic pathway consisting of 13 enzymes for high-yield hydrogen production from starch and water [35]. Building such large systems is extremely challenging; as a result, these articles have received a lot of attention.

### Synthetic Life

Synthetic life is among the most controversial of synthetic biology aims, and has received a lot of attention, even in the mainstream press. Public concerns of possible biological threats resulting from the misuse of these technologies prompted the development of new biosecurity policies [36].

One branch of this field is the *de novo* chemical synthesis and assembly of whole plasmids, viruses and genomes which are then transplanted into host cells. The pX1.0 plasmid is an example of a fully chemically-synthesized plasmid designed by calculating consensus sequences from 8 plasmids [37], while removing genes involved in antibiotic resistance and virulence. The plasmid not only replicated in *E. coli*, but could also self-transfer by conjugation into two other enterobacter species. A chemical synthesis approach was also used to construct whole genomes of bacteriophage G4 (around 10 kilobases in length), resulting in infectious viruses that could pass from one strain of *E. coli* to another [38].

One group has the ambitious long-term aim of building a synthetic chloroplast, and has begun by transplanting photosynthetic bacteria into eukaryotic cells to see whether they can achieve synthetic symbiosis [39]. Remarkably, the authors showed that some cyanobacteria were relatively harmless in zebrafish embryos, compared to *E. coli.* Furthermore, by engineering invasins into the cyanobacteria, they were able to invade and divide inside mammalian macrophages. Synthetic biology is only limited by our imagination, and one can speculate that entire free-living synthetic lifeforms could find their place in the collection in the not-too-distant future.

### Software and Modeling

As the number of biological parts for synthetic biology increases, databases and design methods must evolve. For example, to help researchers search and retrieve biological parts, the Knowledgebase of Standard Biological Parts (SBPkb) is a Semantic Web resource for synthetic biology [40].

The collection also includes two articles presenting Computer Assisted Design software tools. Eugene is a human readable language to specify synthetic biological designs based on biological parts. It also provides a very expressive constraint system to drive the automatic creation of composite parts or devices from a collection of individual parts [41]. Alternatively, the Proto platform also provides a high-level biologically-oriented programming language [42]. Specifications are compiled from regulatory motifs, optimized, then converted into computational simulations for numerical verification.

Ultimately the design tools are only as good as the underlying mathematical models they rely on to make predictions of design behaviors. The collection includes a number of articles applying mathematical modeling approaches rooted in various engineering specialties to the design of synthetic genetic constructs.

Modeling gene networks is at the interface of systems and synthetic biology, and many PLOS ONE modeling papers aim to guide bioengineering projects. A recent example of adapting modeling for re-engineering properties into a system used a standardized synthetic yeast network from the In-vivo Reverse-engineering and Modeling Assessment (IRMA) [43]. Reverse engineering itself was used in a study which ultimately provided guidelines for chemotaxis pathway redesign [44]. Statecharts are used to describe dynamical systems, but have not been applied to gene networks. By doing so explicitly, one study was able to model network motifs and combine them in a complicated interlocked feed-forward loop network [45].

Two-component systems are common regulatory motifs in bacteria, and comprise a kinase that senses environmental signals together with a regulator that mediates the cell response. A recent study asked the question, “what happens if you add a third component that interacts with either of the other two?” [46]. Estimating the parameter space associated with a particular function is very valuable for guiding synthetic engineering approaches, as is determining whether a function is theoretically possible at all. For example, using a geometric argument, it was shown that, surprisingly, even monomer regulators can achieve bistability. This demonstrates the possibility of switch-like behavior in feedback autoloops without resorting to multimer regulators [47].

**Figure 1:**
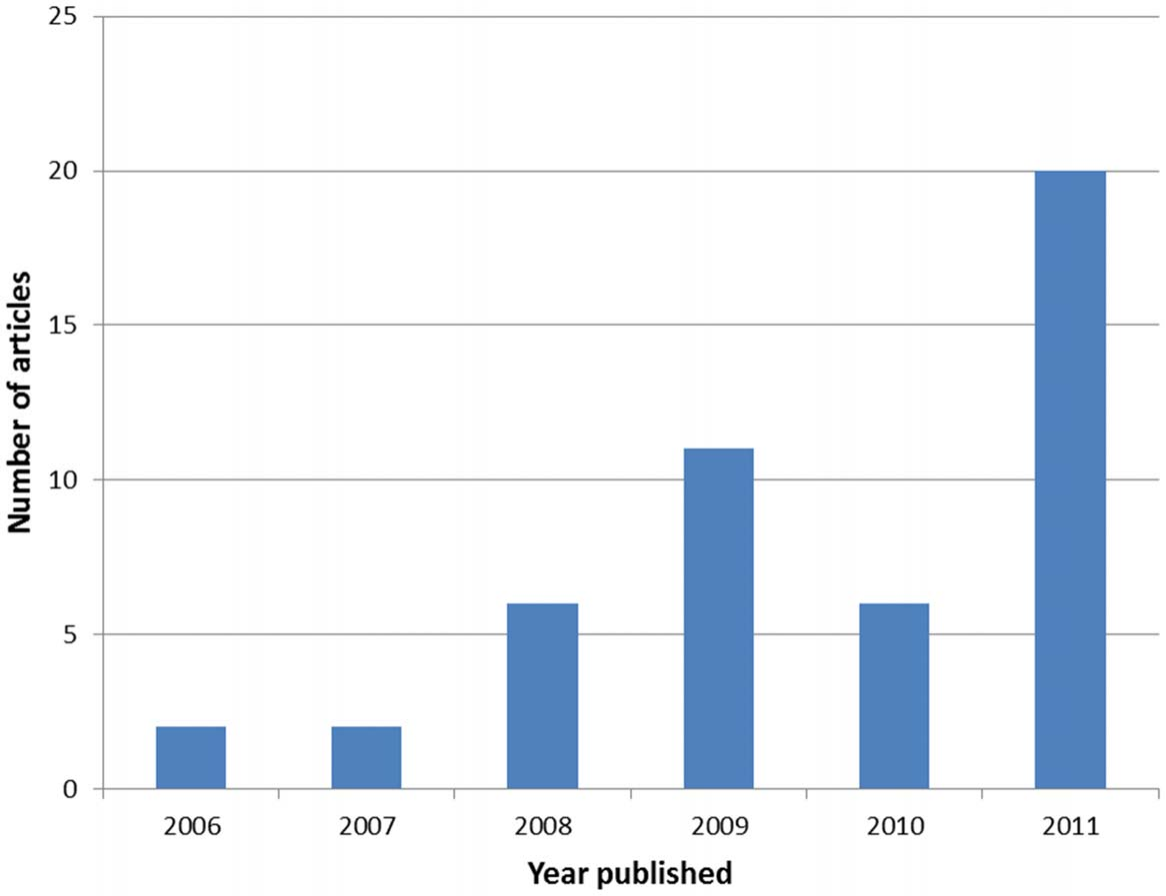
Historical distribution of synthetic biology articles published by PLOS ONE. This figure reports the number of articles in the collection published between 2006 and 2011. It shows a rapid growth of synthetic biology that reflects the growth of the journal and the increased familiarity of synthetic biologists with PLOS ONE.

By combining experiments and computation, one study was able to derive design algorithms for altering synonymous codons in proteins, resulting in drastic expression differences of the same protein sequence [48]. For example, with DNA polymerase and single chain antibodies, expression could be predictably tuned to obtain concentrations ranging from undetectable to 30% of cellular protein. Importantly, using partial least squares regression, the authors noticed that favorable codons were predominantly those read by tRNAs that are most highly charged during amino acid starvation, not codons that are most abundant in highly expressed *E. coli* proteins. This is an important discovery for building genetic constructs that express appropriately inside the target cells.

Computation is a key function of biological networks and several studies in the collection present schemes to achieve this. The first is implemented at the level of chemical reactions and describes functions such as an inverter, an incrementer, a decrementer, a copier, a comparator, a multiplier, an exponentiator, a raise-to-a-power operation, and a logarithm in base two [49]. A key simplification is that the scheme uses only two reaction rates (“fast” and “slow”). A second study models a synthetic gene network to perform frequency multiplication [50]. Both of these studies assume deterministic relationships between input and outputs. Recently, the deterministic assumption has been challenged by experimental and theoretical works analyzing the importance of noise in the dynamics of gene networks [51]. This trend is illustrated in the collection by an article demonstrating that reliable timing of decision-making processes (choosing between multistable states) can be accomplished for large enough population sizes, as long as cells are globally coupled by chemical means [52]. Modeling can often reveal subtle non-intuitive designs, and, as a means of guiding synthetic biology, is likely to become an even larger field in the future.

**Figure 2:**
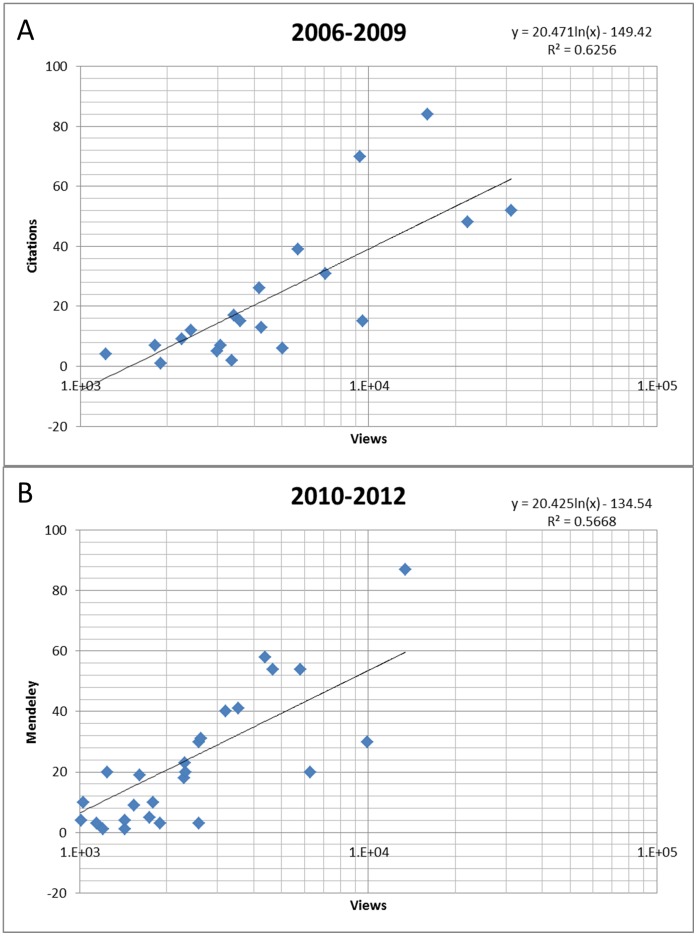
Relationships between article-level metrics. For articles published between 2006 and 2009, there is a positive correlation between the number of times an article is cited in the scientific literature and the number of times it is viewed (A). For articles published between 2010 and 2012, there is a positive relationship between the number of views and the number of citations in the Mendeley social network (B). Metrics, such as number of views and citations in social media, give readers and authors an estimate of the scientific impact of individual articles well before they receive citations in scientific literature.

### Instruments

Nowadays, new technology and machinery is an important driving force for both primary biological discovery and for synthetic biology. A neat example is provided by the use of inkjet printer technology to provide low-cost high-resolution tools; a bacterial piezoelectric inkjet printer was designed to print out different strains of bacteria or chemicals in small droplets onto a flat surface at high resolution [53]. Another group used an inkjet for continuous dosing of diffusible regulators to a gel culture of *E. coli*, allowing 2D spatiotemporal regulation [54]. Precise spatiotemporal control of cells can also be achieved with microfluidics, and a recent report grew dividing yeast cells in a remarkable planar array [55]. Transient pulses of gene expression could be triggered by briefly inducing the GAL1 or MET3 promoters, resulting in coherent induction of cell division across the cell cluster. Other novel culture systems presented in the collection include the development of a 3-D cell culture system using a designer peptide nanofiber scaffold that self-assembled [4]. The peptide could be linked to functional motifs for cell adhesion, differentiation, and bone marrow homing for use with mouse adult neural stem cells.

## The Synthetic Biology Collection: A Dynamic Community Resource

It is remarkable that the collection includes several articles originating from engineers and computer scientists who traditionally publish their work in conference proceedings rather than the journals available to life-scientists. PLOS ONE’s indifference to subject matter made it possible to publish an unprecedented body of articles that reflects the multi-faceted nature of synthetic biology. No less remarkable is the observation that PLOS ONE published several articles originating from iGEM projects [13], [41], [56].

Since 2006, the number of synthetic biology articles published by the journal has been growing steadily (Figure 1). This evolution is consistent with the social trends in synthetic biology that have been mapped in an interesting bibliometric analysis included in the collection [57]. This is an indication that the synthetic biology community is becoming more aware of the services provided by the journal. Looking forward, the collection will make it easier to identify synthetic biology articles among the quickly growing volume of articles published by the journal each day. The content of the collection will be updated periodically as new synthetic biology articles are published by the journal.

Although Journal Impact Factors are a widely-discredited form of evaluating the quality of individual papers, all too often they are still used. Thus, it is imperative to find a better alternative. One of the most exciting features of the PLOS ONE web site is the Metrics tab, displaying article-based metrics that can be used to assess the impact of individual articles. These metrics naturally include traditional indicators, such as the number of citations. The two articles of the collection published in 2006 have been cited 70 and 84 times so far. Almost all the articles published in 2007 and 2008 have received more than 10 citations. The lag between the publication of an article and its citation by others is well known. Fortunately, the Metrics tab also includes more innovative indicators that give the authors and readers alike a real-time estimate of the ‘impact’ of an article. The number of times an article is viewed is an important indicator. Since PLOS ONE is an online journal, all readers view articles online in one way or another. As a result, we hypothesized that the number of times an article was viewed should be a good predictor of the number of citations it will receive. Using data reported in Table S1, we analyzed the relationship between views and citation numbers for articles included in the collection that were published between 2006 and 2009. Figure 2 shows that there is a positive correlation between the two metrics. That relationship does not hold when including more recent articles because of a difference in timing between viewing and citing activities. Articles typically receive a substantial number of views in the first few months after publication, but it takes a few years before they are cited. The 20 articles of the collection published in 2011 have recorded a lot of views, but have not had the time to be cited in the literature yet.

A non-conventional form of citations displayed in the Metrics tab is the number of times an article is bookmarked in social media. We have reported the Mendeley (www.mendeley.com) data in Table S1. Figure 2 shows that there is a positive relationship between the number of views and the number of times articles are bookmarked in this network, at least for the most recent articles of the collection. Older articles are under-represented in Mendeley because this network was not available at the time these articles were published. It will be interesting to see if citations of the collection articles in social media will be a better predictor of citations in the scientific literature than the number of views.

One overarching theme of synthetic biology is standardization [58], [59], which can only be achieved through concerted efforts by members of the community. The field has therefore been deeply influenced by the development of resources such as the Registry of Standard Biological Parts (www.partsregistry.org ). More recently, the development of SBOL, the Open Language for Synthetic Biology (www.sbolstandard.org) illustrates the need to agree on data formats suitable to the development of software tool chains necessary to support experimental efforts. Each article published in PLOS ONE can be the start of a lively conversation. The journal web site provides authors and readers alike with a detailed vision of community connections. The “Share this article” feature allows readers to quickly send an article they find interesting to their networks. The comments tab of the articles provides the community with means to engage in a dialogue focused on specific articles [5], [35], [48], [55]. This feature can also be used by authors to provide updated information about the work presented in the article [13].

When working at its best, science should be an active conversation that keeps refining ideas. We believe that PLOS ONE provides the ideal venue to achieve this, and we hope that the collection will inspire further progress in synthetic biology. Ultimately, we hope that having a clear repository in PLOS ONE should further increase its attractiveness as a home for publishing synthetic biology.

## Supporting Information

Table S1 Article-level statistics for the Synthetic Biology Collection.
(XLSX)
